# Spatial and temporal distribution of rural settlements and influencing mechanisms in Inner Mongolia, China

**DOI:** 10.1371/journal.pone.0277558

**Published:** 2022-11-11

**Authors:** Haitao Zhou, Cuizhen Wang, Yanru Bai, Xiaoli Ning, Shuying Zang

**Affiliations:** 1 Heilongjiang Province Key Laboratory of Geographical Environment Monitoring and Spatial Information Service in Cold Regions, Harbin Normal University, Harbin, China; 2 Baotou Teachers’ College, Baotou, China; 3 Department of Geography, University of South Carolina, Columbia, South Carolina, United States of America; University of Wisconsin Milwaukee, UNITED STATES

## Abstract

Revealing the patterns and influencing mechanisms of spatial and temporal distribution of rural settlements is crucial for rural revitalization and sustainable development. However, our limited understanding of the rural settlements in China’s ethnic minority border areas has hindered the process of their agricultural and rural modernization. Based on data on rural settlements in Inner Mongolia, China in four periods between 1990 and 2020, this study reveals its spatial and temporal distribution characteristics and describes the dynamic transformation process of settlement. Using a geographical detector approach, 17 factors are explored to identify the influencing mechanisms of each factor on the distribution of rural settlements in different regions. The results show obvious regional differences in the spatial distribution of rural settlements in Inner Mongolia, with the largest kernel density values in the west (Hetao irrigation area) and higher kernel densities in the central (Hohhot) and the east (Chifeng and Tongliao). While rural population decreases, rural settlements expand into cultivated land, grassland, and unused land resources. Its spatial distribution is significantly influenced by the factors of distance to cultivated land, distance to towns, and population density. The east of the study area is mainly controlled by temperature, while vegetation type and vegetation coverage have a greater impact in the west. The interactions between two influencing factors possess bilinear or nonlinear enhancement relationships. This study enriches the understanding of the rural settlements in ethnic minority border areas, which provide reference for the improvement of rural human settlement environment in Inner Mongolia.

## 1. Introduction

Rural settlements are an important living space for the rural population [1–3]. Spatial and temporal distribution patterns reveal the footprints of human-land interaction and the coupling process of changes in the human-land relationship [4, 5]. Meanwhile, as the core for grasping adjustments to the rural human-land relationship, spatial and temporal patterns are indispensable to current status and evolution of rural revitalization [6–8]. With the rapid social and economic development of China’s reform and opening up, predominantly rapid urbanization and industrialization, China’s rural settlements present a series of “rural diseases” such as intensified land use, lack of planning, hollowing out, and environmental defilement, which have seriously limited the modernization of agriculture and rural areas [9–13]. To solve this problem, exploring the spatial and temporal distribution characteristics of rural settlements and revealing the laws of their diachronic evolution, including the influencing mechanisms, have become the current research hot spots.

Rural settlements are the regional carriers of natural, economic, and social composite systems [14–16]. Spatial distribution and agglomeration of rural settlements on different scales and landforms have obvious regional differences [2, 17]. In China, rural settlements show an overall low degree of agglomeration, small scale, and large regional variation [1, 2, 18]. For example, the density of settlements in southeast coastal areas is higher than that in northwest inland, while plain areas are higher than mountainous and hilly areas, traditional agricultural farming areas are higher than pastoral areas, and economically developed areas have a higher density than economically underdeveloped areas [2, 17–21]. The spatial pattern of rural settlements in plain areas is mainly random and dispersed, while in cold alpine areas and desert fringes settlements are mainly clustered [2]. Oasis agricultural areas, too, are small and scattered [21].

In recent years, China’s rural areas have experienced the phenomenon of population decrease and land use increase [22–24]—that is, the rural population is decreasing due to urbanization, but the area occupied by rural settlements is increasing. The increase in rural settlements area is mainly dominated by the occupation of cultivated land, but there are also forest, grassland, and unused land types [4, 23, 25–27]. The occupation of cultivated land by rural settlements not only poses a threat to national food security, but also damages the surrounding environment and biodiversity [21, 26]. The factors influencing the distribution of rural settlements differ at different research scales. At the national scale, geomorphology, climate, and population distribution are the main influencing factors, while on a smaller scale, such as the county, the main influencing factors may be slope, aspect, or distribution of water resources [28–31]. The degree of influence of factors on the spatial distribution of settlements in different periods can also change. The natural environment was the main factor for the selection of settlement locations in the early stage [32], with the topography playing a controlling role [28], while the distribution of water resources and cultivated land and population density also influenced the spatial distribution of settlement location [33–36]. With rapid social and economic development, the role of natural environmental constraints gradually declines, and the impact of urban centers, resource areas with ornamental recreation, convenient transportation, and free trade development in rural settlements becomes increasingly obvious [28, 32, 37]. Infrastructure construction, government policies and systems, and local ethnic customs and culture also play an important role in the spatial distribution of rural settlements [38–40]. We thus know that the spatial and temporal distribution of rural settlements and the spatial and temporal effects of influencing factors depend on scale and time. Nonetheless, research on the evolution in space and time of ethnic minority rural residential areas and their influencing factors is limited, especially in the Inner Mongolia autonomous region of the minority frontier area in northern China.

Inner Mongolia is located in a fragile ecological environment in the farming-pastoral ecotone in northern China, where the geomorphological types are complex and diverse; the natural environment widely varies; the environmental evolution is obvious and drastic; and climate difference is significant [41, 42]. It has always been a multi-cultural, multi-ethnic, and multi-religious community, with a vast territory that is sparsely populated and shows uneven regional economic development [20, 31]. Due to the influence of multiple factors such as natural environment, location conditions, production and lifestyle, ethnic customs, and religious beliefs, as well as the lack of rural planning guidance, the region’s rural residential space layout has long been in a state of spontaneous disorder, which has caused widespread problems such as extensive settlement land and scattered layout, and has also lead to tension between people and land while seriously restricting the development of the region. To improve the ecological environment, the Chinese government began to implement the “Three-north shelterbelt system project” in 1978, along with the “Beijing-Tianjin sandstorm source control project,” the “natural resources protection project in key state-owned forest areas,” and the “project of returning farmland to forest and grassland”, with Inner Mongolia as the main site for these projects [41, 42]. As a result, the region has witnessed dramatic changes in the spatial layout of its rural settlements.

In recent years, Inner Mongolia has been planning to construct an important ecological security barrier in northern China. Additionally, a series of major livelihood projects began to implement, such as immigration and relocation in ecologically vulnerable areas, beautiful countryside, characteristic towns, and comprehensive land improvement. The “top-down” immigration policy led by the government, which relocates residents from a deteriorating ecological area to concentrated resettlement, has not achieved a real sense of moving out, stabilizing, and enriching [5, 9, 43]. It is therefore necessary to fully understand the spatial distribution characteristics and influencing mechanisms of rural settlements in different periods, scales, and regions in Inner Mongolia to put forward a feasible policy system according to local conditions and achieve the win-win goal of constructing an ecological civilization and ensuring people’s living and working in peace and contentment in the new era. This poses the following questions. What are the characteristics of the spatial distribution of rural settlements in different periods, scales, and regions of Inner Mongolia? Does the phenomenon of population decrease and land use increase occur in rural areas of Inner Mongolia, and where does residential land come from? What are the main factors affecting the evolution of settlements? How do the influencing factors interact with each other?

The existing studies have mainly considered the pattern and influencing factors of rural settlements for a certain year within a county administrative region of Inner Mongolia. For example, based on the landscape data for Zhenglan Banner in 2005, used landscape metrics to indicate the spatial pattern of human settlements and analyzed the relationship between the location of settlements and their DEM and slope, NDVI, and the distance to rivers, and roads [20]. Some studies also selected Kangbashi New Area and Zalut Banner to represent urban and rural settlements, respectively, and conducted a comparative study using settlement data in 2006 and 2015 years to reveal their spatial evolution characteristics [31]. while assessment of different time periods and different scales in different areas of Inner Mongolia are nearly nonexistent.

Based on the above questions, according to the principle of comprehensive geographical division, Inner Mongolia is divided into eastern, central and western regions, this study adopted the data from rural settlements in Inner Mongolia from the years 1990, 2000, 2010, and 2020 to systematically reveal their spatial and temporal distribution characteristics. The dynamic transformation process of rural settlement land is elucidated by employing the land type transfer matrix method. Seventeen influencing factors were selected related to the natural environment, location conditions, ecological environment, and socioeconomic aspects. A geographic detector approach was utilized to clarify how each influencing factor affects the spatial distribution of settlements for different scales and regions. The importance of each factor was identified, and the interaction mechanism between factors was explained to provide theoretical support to promote high-quality development and optimal regulation of rural settlements in Inner Mongolia.

## 2. Materials and methods

### 2.1 Study area

**Fig 1:**
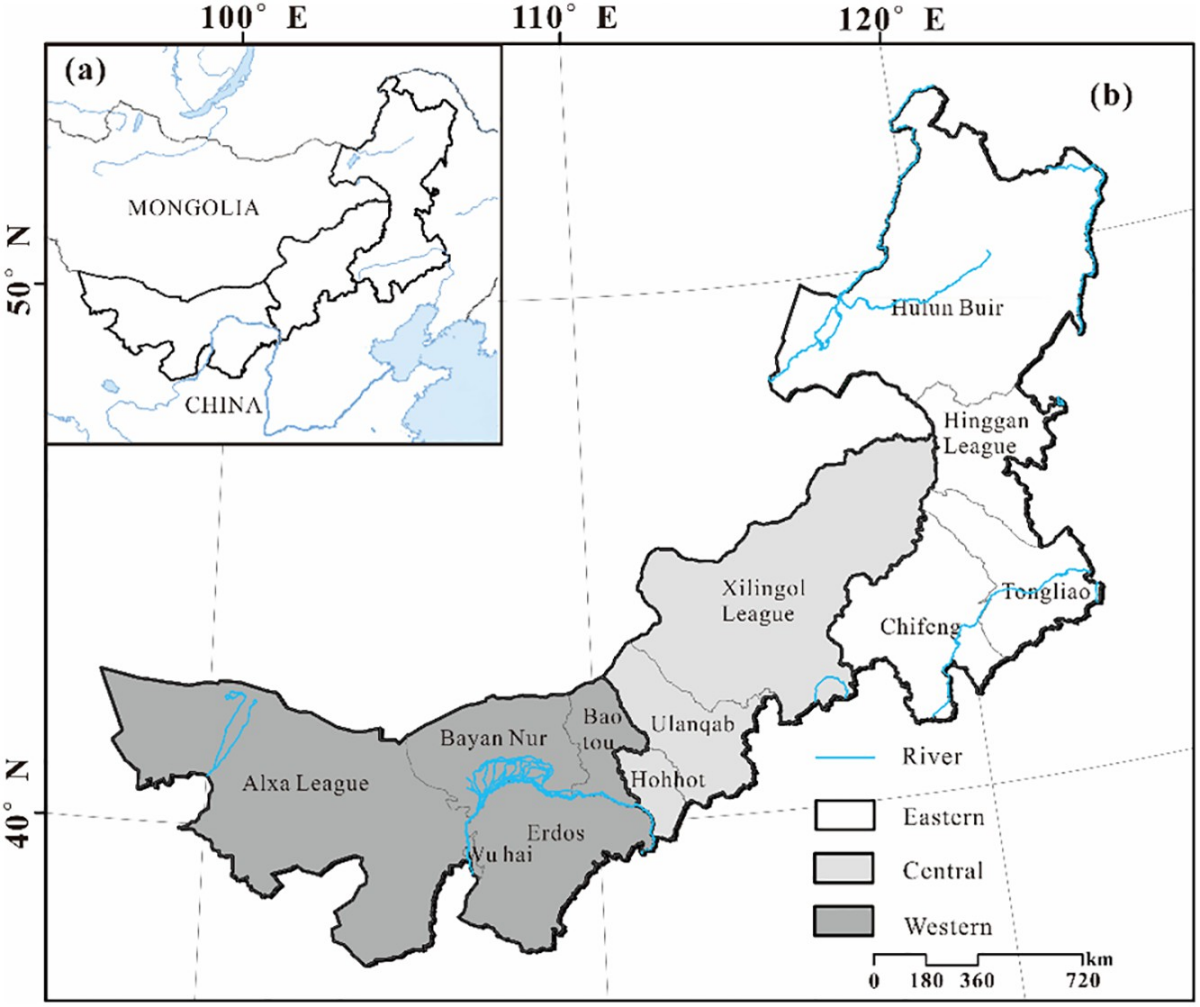
Location and administrative divisions of the study area. (a) Location of Inner Mongolia in China; (b) Administrative divisions of the Inner Mongolia. The basemap is form United States Geological Survey (https://apps.nationalmap.gov/services/), cartographic software: ESRI. ArcGIS.

The Inner Mongolia Autonomous Region is located in the border area of ethnic minorities in the north of China (Fig 1(a)) at 97°12′E–126°04′E, 37°24′N–53°23′N, covering an area of 1183000 km^2^. It is the third-largest province in China. It extends obliquely from northeast to southwest, with a narrow east-west length of about 2400 km and a maximum north-south span of about 1700 km. The landform is mainly plateau, with elevations ranging from 89–3387 m and most areas above 1000 m. According to the principle of comprehensive geographical division, Inner Mongolia is divided into eastern (Hulun Buir, Hinggan League, Tongliao, and Chifeng), central (Hohhot, Ulanqab, and Xilingol League) and western regions (Baotou, Erdos, Wuhai, Bayan Nur, and Alxa League) (Fig 1(b)). Affected by geographical location and topography, the region has mainly a temperate continental monsoon climate, with obvious climatic differences between the eastern, central, and western regions [44]. The average temperature is 25°C in summer, and the average minimum temperature in the central and western regions is lower than −20°C in winter, while in the eastern forest region it is lower than −50°C. The landscape transition from east to west is visible, showing, in order, a forest-grassland-desert landscape [45, 46], spanning pastoral areas, farming-pastoral ecotone, and farming areas from north to south. The population consists of 55 ethnic groups, including Mongolian and Han. The seventh census data showed that the urban resident population in Inner Mongolia increased from 1990 to 2020, and the rural population continued to decrease (http://tj.nmg.gov.cn/). During the “13th Five-Year Plan” period, Inner Mongolia completed the relocation of 1,249,000 poor people and built 533,000 housing units (https://www.nmg.gov.cn/). The spatial layout of rural settlements has undergone profound changes, but some resettled households have returned, resulting in a waste of land and other resources.

### 2.2 Data sources and processing

Multi-source datasets were involved in this study (Table 1). Four periods of land use type data for Inner Mongolia from 1990 to 2020 were derived from the Resource and Environment Science and Data Center of the Chinese Academy of Sciences (https://www.resdc.cn). Seven land use types were explored in this study: cultivated land, forest land, grassland, water area, construction land, unused land, and rural settlements. The vegetation and soil type data were drawn from the 1:1,000,000 vegetation map of China, and the 1:1,000,000 soil map of China, respectively (https://www.resdc.cn). Digital elevation model (DEM) data, with a spatial resolution of 30 m, were obtained from the Geospatial data cloud (https://www.gscloud.cn/). Slope and aspect were generated with the DEM using ArcGIS10.2. Temperature and precipitation of 96 meteorological stations in Inner Mongolia from 1990 to 2020 were downloaded from the China Meteorological Science Data Center (http://data.cma.cn). Road and railway data came from the 1:1,000,000 national basic geographic database of the National Geomatics Center of China (http://www.ngcc.cn/ngcc/). The vegetation coverage data were derived from the Geographic Information monitoring cloud platform (http://www.dsac.cn/), with a spatial resolution of 1 km. In addition, Point of Interest (POI) data mainly including town, school, and hospital data. The population and Gross Domestic Product (GDP) data were obtained from the statistical Yearbook and Statistical Bulletin of Inner Mongolia. All data layers were resampled to a unified grid size of 1 km.

**Table 1:** Description of the data used in this study.

Data	Time	Type	Resolution	Source
Land use type	1990,2000,2010,2020	Raster	1 km	Resource and Environment Science and Data Center of the Chinese Academy of Sciences (https://www.resdc.cn)
Vegetation type map	2001	Raster	1:000000	
Soil types	1995	Raster	1:000000	
DEM	2020	Raster	30 m	Geospatial data cloud (https://www.gscloud.cn/)
Temperature, precipitation	1990~2020		96 points	China Meteorological Science Data Center (http://data.cma.cn)
Vegetation coverage	2020	Raster	1 km	Geographic Information monitoring cloud platform (http://www.dsac.cn/)
POI Data	2020		points	Amap (https://ditu.amap.com/)
Road, railway	2020	Vector	1:000000	National Geomatics Center of China (http://www.ngcc.cn/ngcc/)
Statistical Yearbook	2021			http://tj.nmg.gov.cn/

### 2.3 Methods

#### 2.3.1 Kernel density analysis

Kernel density analysis is commonly used to describe the spatial distribution density of point elements to visually express their spatial distribution characteristics [22, 28]. The formula is as follows:

$$f\left(\text{x},\;\text{y}\right)=\frac{1}{nh}\overset{n}{\underset{i=1}{\sum }}\text{k}\left(\frac{x-x_{i}}{h}\right)$$ (1)

Where *f (x*, *y)* denotes the density estimate located at position (x, y); *n* is the number of rural settlements; *h* is the bandwidth, that is, the search radius distance; *K* is the kernel density function, and (***x*** − ***x***_***i***_) is the distance between two rural settlements.

#### 2.3.2 Spatial autocorrelation

Spatial autocorrelation can reveal the agglomeration pattern of the spatial distribution of geographical elements [47]. The Moran’s index (Moran’s I) value is calculated to evaluate the visibility of the index, which was [–1, 1]. When Moran’s I >0 indicates a positive correlation within the space, which means that the spatial distribution pattern of geographical elements is clustered and the larger the value is, the more obvious the cluster. In contrast, Moran’s I <0 indicates a negative correlation, in this case, means uniform distributions. In addition, if Moran’s I = 0, the representation spatial distribution is random.

#### 2.3.3 Transfer matrix

The transfer matrix reflects the number of areas where rural settlement land is transformed into other land types within a certain period of time, and the amount of land converted from other land types to settlement land. Firstly, ArcGIS10.2 is used to generate the land use transfer matrix, and the quantity of land transferred between different types from 1990 to 2020 is obtained. Then, the transfer trend is represented by a Sankey diagram, which intuitively represents the transformation trend characteristics of rural settlement land types and the differences between different land use types.

#### 2.3.4 Geographic detector

Geographic detector, a group of statistical methods, is mainly applied to explore the spatial differentiation of various phenomena to reveal their influencing factors, and multi-factor interactions [48]. It provides a better expression of the similarities in the same area and the differences in different areas, moreover, it explains the explanatory strength of the independent variable X to the dependent variable Y [22]. Factor detection determines the extent to which a factor explains the spatial differentiation of variables. The spatial distribution layer of rural settlements in Inner Mongolia was overlaid with each influencing factor layer to identify the importance of the influencing factors. The computational formula is as follows:

$$p_{X,Y}=1-\frac{\sum_{\text{h}=1}^{\text{L}}{\text{N}}_{\text{h}}\sigma_{\text{h}}^{2}}{\text{N}\sigma^{2}}$$ (2)

where *p*_*X*,*Y*_ is the explanatory power of influencing factor *X* to rural settlement distribution *Y*, *N* is the number of regions, *L* is the number of influencing factors, and ***N***_***h***_ and $\sigma_{\text{h}}^{2}$ are the sample size of *h* layer and the variance of rural settlement, respectively. The value of *p*_*X*,*Y*_ is strictly within [0, 1] and the larger the value, the stronger the explanatory power of influencing factor *X* to the rural settlement. Previous studies have shown that the spatial distribution of rural settlements is mainly affected by the basic geographical conditions, traffic conditions, farming conditions, infrastructure construction, ecological environment and social and economic conditions [28–32]. Combined with the situation of the research area, we selected 17 factors from the above 7 aspects (Table 2): DEM (X_1_), slope (X_2_), aspect (X_3_), distance to towns (X_4_), distance to roads (X_5_), distance to railway (X_6_), distance to hospitals (X_7_), distance to schools (X_8_), distance to water (X_9_), distance to cultivated land (X_10_), temperature (X_11_), precipitation (X_12_), Soil types (X_13_), vegetation types (X_14_), vegetation coverage (X_15_), per capital GDP (X_16_), and population density (X_17_).

**Table 2:** Impact factor.

Category	Factor
Basic geographical conditions	DEM (X1); Slope (X2); Aspect (X3)
Traffic conditions	Distance to towns (X4); Distance to roads (X5); Distance to railway (X6)
Education and health infrastructure	Distance to hospitals (X7); Distance to schools(X8)
Farming conditions	Distance to water (X9); Distance to cultivated land (X10)
Climate conditions	Temperature (X11); Precipitation (X12)
Ecological environment	Soil types (X13); Vegetation types (X14); Vegetation coverage (X15)
Social and economic conditions	Per capital GDP (X16); Population density (X17)

Interaction detection is used to reveal whether any pair of factors X_n_ and X_m_ have an interactive influence on the rural settlement, primarily appearing in five types, as shown in Table 3.

**Table 3:** Interaction types.

Interaction	Criterion
Nonlinear weakening	*Xn ∩ Xm < Min (Xn*, *Xm)*
Unilinear reduction	*Min (Xn*, *Xm) < Xn ∩ Xm < Max (Xn*, *Xm)*
Bilinear enhancement	*Xn ∩ Xm > Max (Xn*, *Xm)*
Mutual independence	*Xn ∩ Xm = Xn + Xm*
Nonlinear enhancement	*Xn ∩ Xm > Xn + Xm*

## 3. Results

### 3.1 Spatial distribution characteristics

**Fig 2:**
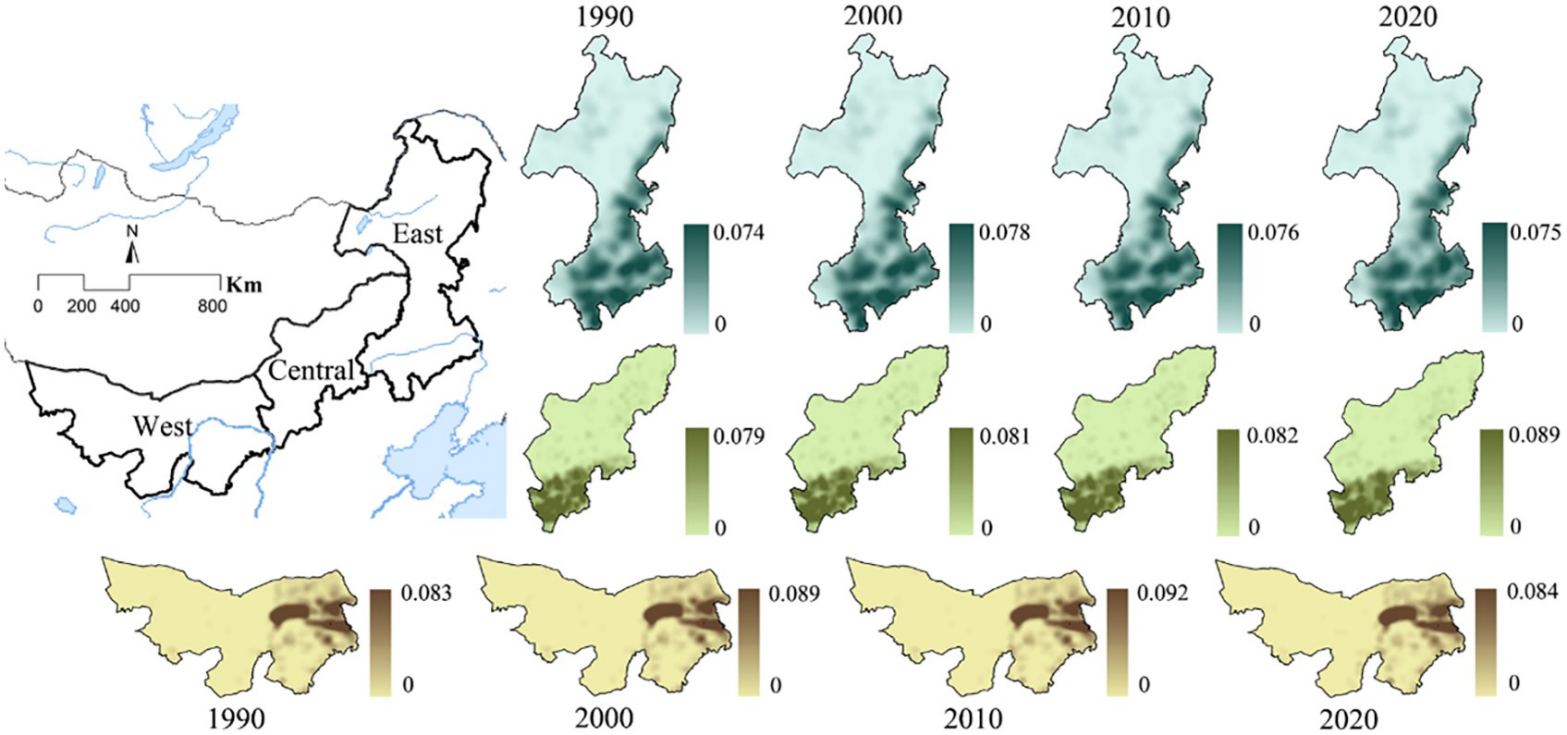
Kernel density maps of rural settlements in Inner Mongolia in 1990–2020. The basemap is form United States Geological Survey (https://apps.nationalmap.gov/services/), cartographic software: ESRI. ArcGIS and Adobe photoshop CC.

The spatial distribution of rural settlements in Inner Mongolia shows significant regional difference according to kernel density values (Fig 2). The spatial distribution generally presents a northeast-southwest direction. The highest peak value appeared in the Hetao irrigation area in the west of Inner Mongolia and was significantly higher than Hohhot in the central region and Chifeng and Tongliao in the east. However, during the period 1990–2020, the kernel density value of rural settlements in the western region showed first an increase and then a decrease, while the central and eastern regions showed a gradual increase, with a larger increase in the central region, especially during the period 2010–2020. The kernel density values for rural residential areas in Alxa League in the western part of Inner Mongolia, Xilingol League in the central part, and Hulun Buir in the northern part are always low, especially in Alxa League. From the north-south direction, the kernel density in the southern agricultural area was significantly higher than that in the northern pastoral area.

The spatial distribution pattern of rural settlements in Inner Mongolia is predominantly characterized by clustering, with obvious regional differences (Table 4). The degree of rural settlement clustering is highest in the east, followed by the central area, and is relatively low in the west. In the same region, the degree of clustering also changes for different years. At the overall scale of Inner Mongolia, the degree of clustering is V-shaped, decreasing first and then increasing, with the lowest index value of 0.808 in 2010, which increases to 0.836 in 2020. In the eastern and central regions, the clustering showed an inverted V-shape, which increased first and then decreased, but the peak inflection point was not consistent. The maximum value of 0.923 in the eastern region in 2010 was the inflection point, while for the central region 2000 was the inflection point with a peak value of 0.682. The degree of clustering in the western region has been increasing but is lower than in the other regions.

**Table 4:** Moran’s index of rural settlements in Inner Mongolia.

Year	Inner Mongolia	Eastern region	Central region	Western region
1990	0.822	0.905	0.669	0.362
2000	0.817	0.911	0.682	0.363
2010	0.808	0.923	0.676	0.367
2020	0.836	0.907	0.675	0.422

### 3.2 Temporal trends of population and land use

**Fig 3:**
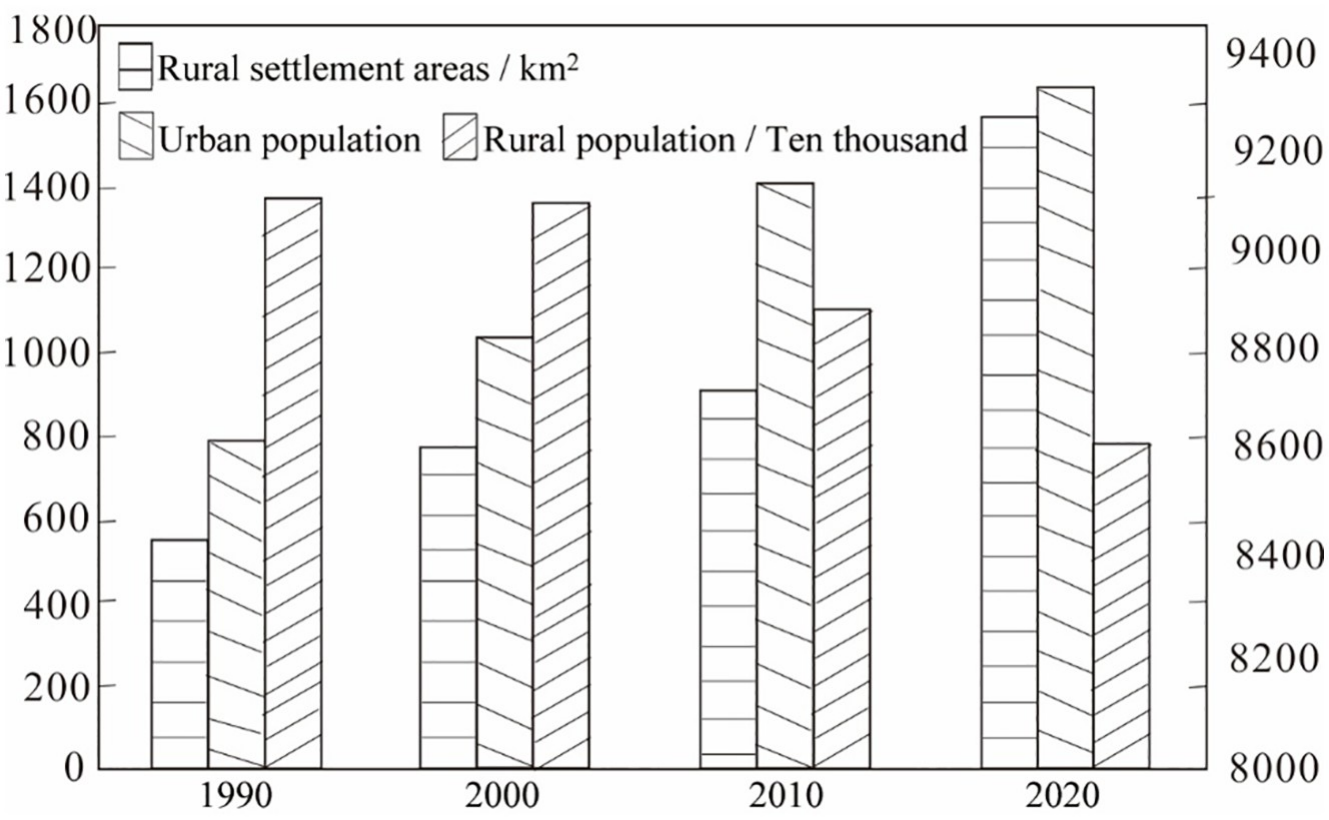
Change of urban population, rural population, and rural settlement land in Inner Mongolia during 1990–2020.

During the period 1990–2020, the area of rural settlements in Inner Mongolia showed an increasing trend, from 8,438.52 km^2^ in 1990 to 9,208.79 km^2^ in 2020 (Fig 3). The area of rural settlements increased by 159.93 km^2^ from 1990 to 2000, with a rate of increase greater than that from 2000 to 2010, but lower than from 2010 to 2020. The expansion of rural settlements in Inner Mongolia was the greatest and the fastest during the period 2010–2020. The latest data from the seventh population census show that the rural population of Inner Mongolia has been decreasing from 1990 to 2020. In 1990, the rural resident population of Inner Mongolia was 13.6596 million, which decreased to 7.82117 million in 2020, a decrease of 5.83779 million (42.7%). This indicates that the rural areas of Inner Mongolia have been experiencing a population decrease and land use increase, and during the period 2010–2020, the settlement area expanded the most and the population decreased the most.

### 3.3 Conversion of land use for rural settlement

**Fig 4:**
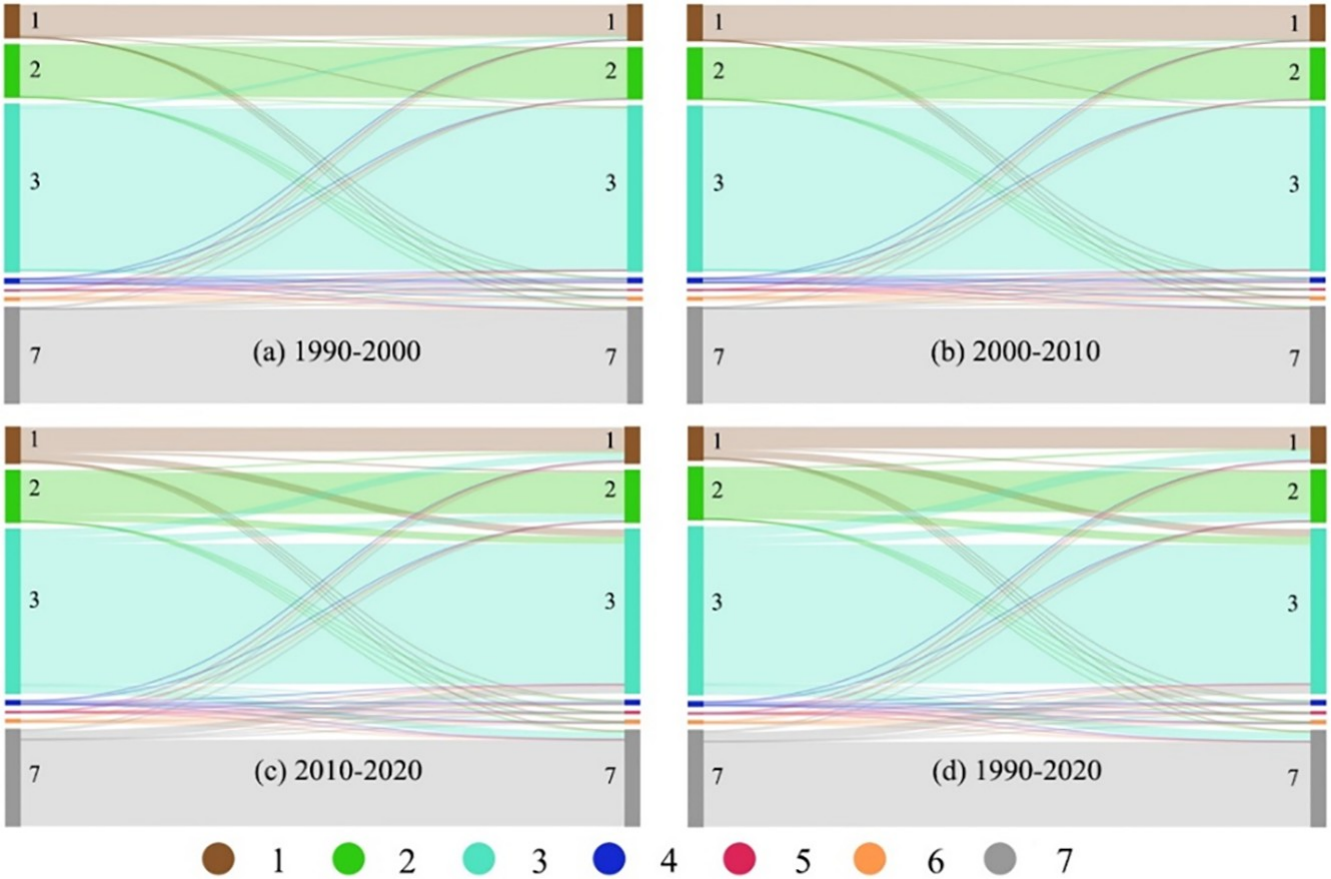
Transition trends of the land use type at a decadal interval in four periods. Note: 1: cultivated land, 2: forest land, 3: grassland, 4: water area, 5: construction land except rural settlement land, 6: rural settlement land, and 7: unused land.

During the period 1990–2000, settlement expansion mainly occurred on cultivated land and grassland, with an area of 149.20 km^2^ and 121.20 km^2^, respectively (Table 5). Unused land, forest land, and water areas were also occupied, but the proportion was relatively low (Fig 4(a)). During 2000–2010 and 2010–2020, cultivated and grassland remained the main sources of land for settlement expansion. In all three periods, the area occupied by cultivated land was the largest, but the increase in the area of land of different types encroached upon by settlement expansion did not follow the same proportions in different periods. The proportional increase of encroached grassland was greater than that of encroached cultivated land during 2000–2010 (Fig 4(b)), but the cultivated land encroachment was largest between 2010 and 2020 (Fig 4(c)). From 1990 to 2020, the main sources of land for rural expansion in Inner Mongolia were cultivated land, grassland, and unused land, while the conversion of residential land to cultivated land, grassland, and unused land, also appeared, but the conversion ratio was low (Fig 4(d)).

**Table 5:** Land use type transfer matrix.

2000	1	2	3	4	5	6	7
1990							
1	100157.02	426.71	4303.86	132.51	22.82	149.20	429.20
2	2423.99	162425.06	2966.30	10.54	1.37	8.27	59.08
3	11041.69	1328.92	518574.73	338.42	44.80	121.20	5217.37
4	219.90	16.93	190.59	12692.67	1.20	5.18	348.55
5	4.93	0.39	4.22	0.21	1475.51	0	0.54
6	116.26	0.74	24.86	0	0.75	8290.17	1.75
7	831.76	109.21	3222.47	702.96	0.61	20.44	302350.43
2010	1	2	3	4	5	6	7
2000							
1	111566.03	646.58	2010.28	130.56	81.93	154.07	206.11
2	327.95	162907.45	877.47	47.52	15.93	10.00	121.65
3	2642.66	1997.80	520041.41	309.07	230.24	138.32	3927.54
4	179.40	32.42	280.01	12380.18	3.78	4.92	996.61
5	4.65	1.52	5.33	1.03	1532.35	1.25	0.92
6	96.62	9.55	55.50	9.26	22.44	8386.86	14.23
7	361.88	95.07	3061.64	254.86	34.11	7.25	304592.11
2020	1	2	3	4	5	6	7
2010							
1	77667.41	6058.85	22905.64	1291.15	817.16	3517.29	2921.69
2	5903.74	133003.76	23871.39	369.18	170.43	309.29	2062.58
3	23432.63	23724.32	448303.14	2791.27	1827.03	2590.21	23663.04
4	1199.92	354.20	2483.26	7547.80	73.26	128.86	1345.17
5	190.30	65.96	245.81	27.16	1316.25	28.64	46.67
6	3416.85	321.67	2183.92	163.50	80.46	2086.08	450.18
7	3141.99	2401.49	25355.16	1520.51	580.89	548.42	276310.71
2020	1	2	3	4	5	6	7
1990							
1	68509.10	5294.95	23427.74	1354.22	871.05	3465.52	2698.75
2	7209.41	132161.96	25677.13	372.58	162.14	298.57	2012.83
3	30702.25	25307.05	443924.39	2971.21	2060.22	2755.65	28946.38
4	1395.41	371.02	2607.23	7138.20	68.23	133.55	1761.38
5	129.16	55.06	169.94	21.62	1054.05	20.34	35.63
6	3341.05	313.85	2110.05	163.35	74.75	1992.22	439.25
7	3666.46	2426.37	27431.85	1689.40	575.05	542.95	270905.81

Note: 1: cultivated land, 2: forest land, 3: grassland, 4: water area, 5: construction land except rural settlement land, 6: rural settlement land, and 7: unused land. Area: km^2^.

### 3.4 Influencing mechanism analysis

**Fig 5:**
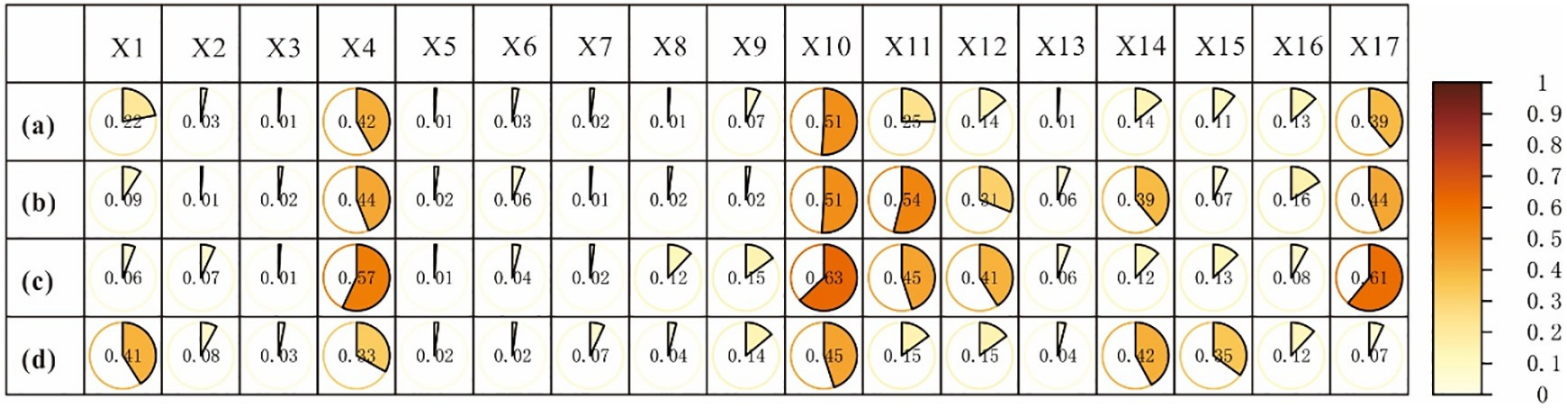
Influencing factors on rural settlement. Note: (a) Inner Mongolia, (b) Eastern region of Inner Mongolia, (c) Central region of Inner Mongolia, and (d) Western region of Inner Mongolia. X_1_: DEM, X_2_: slope, X_3_: aspect, X_4_: distance to towns, X_5_: distance to roads, X_6_: distance to railway; X_7_: distance to hospitals, X_8_: distance to schools, X_9_: distance to water, X_10_: distance to cultivated land, X_11_: temperature, X_12_: precipitation, X_13_: Soil types, X_14_: vegetation types, X_15_: vegetation coverage, X_16_: per capital GDP, and X_17_: population density.

The factor detector revealed the influence of each factor on rural settlements. As shown in Fig 5(a), the pivotal factors affecting the spatial distribution of rural settlements in Inner Mongolia is the distance to cultivated land (0.51), distance to towns (0.42), and population density (0.39). These three factors are also important factors influencing settlement in eastern Inner Mongolia, and their explanatory power has increased. However, the temperature has the greatest explanatory power for the spatial distribution of rural settlements in the east, with a P value of 0.54 (Fig 5(b)). Meanwhile, the explanatory power of the factors precipitation and vegetation type increased significantly. In the central region, the distance to cultivated land, population density, and distance to towns factors have the strongest explanatory power, with maximum values of 0.63, 0.61, and 0.57, respectively, but the temperature factor is less influential than in the eastern region. Although the distance to cultivated land was also the most important factor affecting the spatial distribution of settlements in the west, the influence value has decreased. The P values for distance to towns and population density decreased, and that for population density value decreased by only 0.07. The explanatory power of DEM, vegetation type, and vegetation cover factors increased (Fig 5(d)). It can be seen that the importance of factors affecting the spatial distribution of rural settlements varies at different scales, and the importance of the same factor is not consistent across different areas on the same scale.

**Fig 6:**
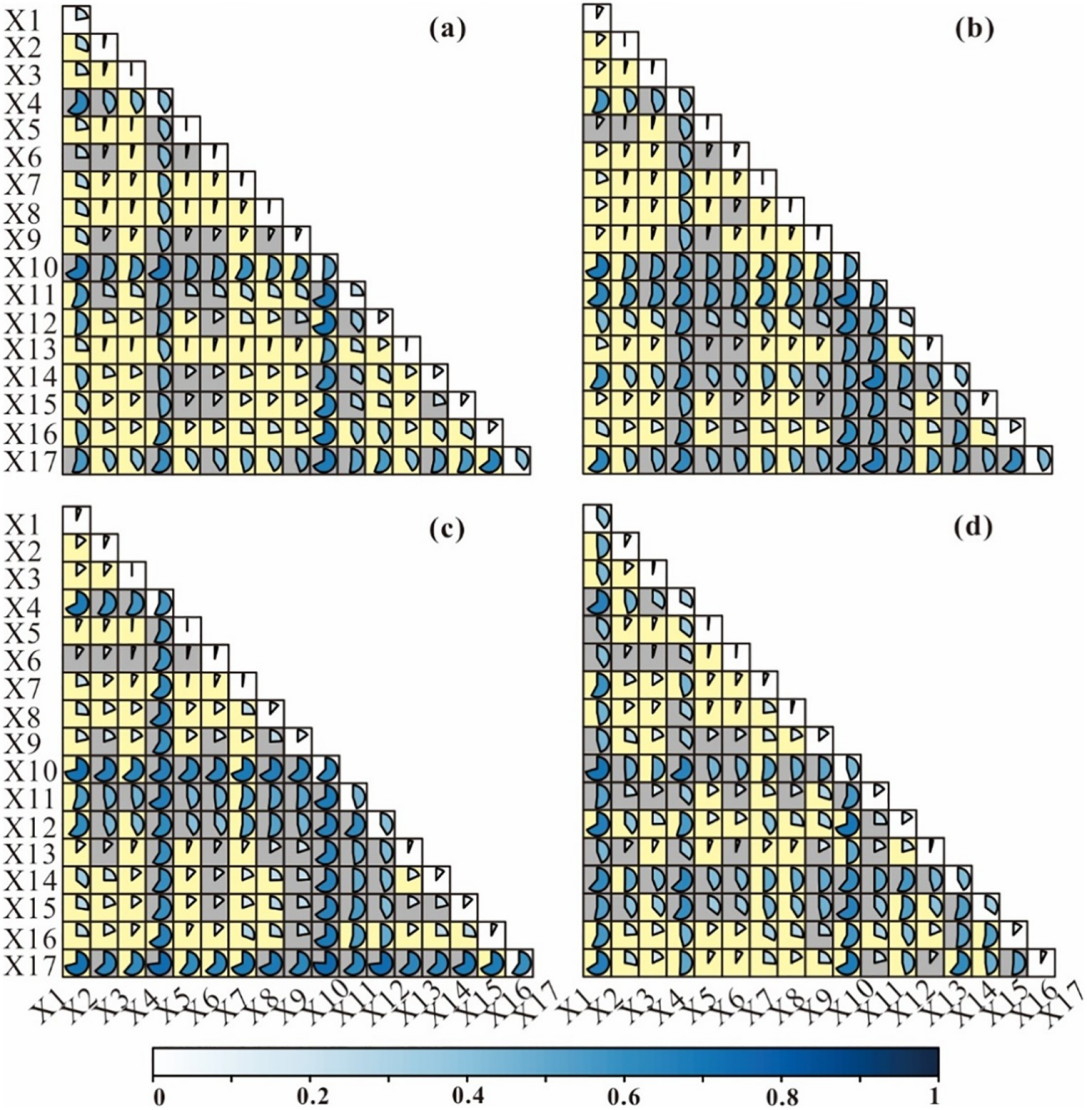
Interaction of influencing factors on rural settlement. Note: (a) Inner Mongolia, (b) Eastern region of Inner Mongolia, (c) Central region of Inner Mongolia, and (d) Western region of Inner Mongolia. X_1_: DEM, X_2_: slope, X_3_: aspect, X_4_: distance to towns, X_5_: distance to roads, X_6_: distance to railway; X_7_: distance to hospitals, X_8_: distance to schools, X_9_: distance to water, X_10_: distance to cultivated land, X_11_: temperature, X_12_: precipitation, X_13_: Soil types, X_14_: vegetation types, X_15_: vegetation coverage, X_16_: per capital GDP, and X_17_: population density. The yellow background denotes nonlinear enhancement, and the gray denotes bilinear enhancement.

Interaction detection reveals the explanatory power of influencing factors under paired interaction to assess whether they are enhanced, weakened, or independent. As demonstrated in Fig 6, the interaction of influencing factors for rural settlements had an enhancement relationship, not a simple superposition effect, but a bilinear or nonlinear enhancement, and there were no independent or weakening relationships. On the scale of the whole study area (Fig 6(a)), the interaction between the X_10_ and X_12_ factors was the strongest, with an interaction value of 0.70. The interaction values between X_10_ and X_11_, and X_10_ and X_16_ were 0.68. The strongest interaction appeared between X_10_ and X_1_ in the eastern region (Fig 6(b)), followed by the interaction be-tween X_10_ and X_11_ at 0.68. The two-factor interaction between X_10_ and X_17_ was the strongest in the central region, although X_17_ and X_4_ and X_17_ and X_12_ were also crucial (Fig 6(c)). In contrast, in the western region, the interaction between X_10_ and X_12_, X_10_ and X_1_ had the strongest effect (Fig 6(d)).

## 4. Discussion

This study revealed that the agglomeration density of rural residential areas in Inner Mongolia was the largest in Hetao irrigation area, which is consistent with past studies [2, 49]. The Hetao irrigation area is an alluvial plain irrigated by the Yellow River, with flat terrain, fertile land, and crisscross channels [50]. It is the largest primary irrigation area in Asia and one of the three super large irrigation areas in China, as well as an important commercial grain and oil production base for the autonomous region and the country as a whole. The density of rural settlements in Hetao showed an increasing trend from 1990 to 2010, then decreased in 2020. The main reason for this may be that Hetao region began to implement 666.67 km^2^ of land consolidation, 3333.35 km^2^ of low-yielding farmland transformation, water-saving irrigation, and other large-scale agriculture-related construction projects in 2011, and comprehensively started the implementation of rural land renovation projects with homestead replacement, village layout adjustment, and farmers’ housing transformation.

In the central region, Hohhot has a high density of rural settlements. Hohhot is the capital of the Inner Mongolia Autonomous Region and its political, economic, cultural, scientific, educational, and financial center. Together with the cities of Baotou and Erdos, it constitutes the most dynamic advantaged region of Inner Mongolia, with convenient transportation and better infrastructure facilities. The high-value density of rural settlements in the eastern region appears primarily in the area of Chifeng and Tongliao. The eastern region has relatively low topography, more fertile and contiguous arable land, and more developed agricultural and livestock production. The third land survey data showed that the cultivated land in Tongliao, Hulun Buir, Chifeng, and Hinggan League accounted for 66% of the cultivated land in the autonomous region (https://www.nmg.gov.cn/). The Hulun Buir region in the northeast has a lower population due to its cold climate, and the Alxa League in the west is also sparsely populated. The natural environment of Alxa League is a relatively harsh, sandy desert, and the ecological environment is fragile, making it unsuitable for human habitation [41, 51].

The overall spatial distribution of rural settlements in Inner Mongolia follows a northeast-southwest direction, and the density of rural settlements in southern agricultural areas is significantly higher than in northern pastoral areas. This phenomenon is closely related to population distribution. Historically, the population distribution in Inner Mongolia is extremely uneven, with a sparse population on the plateau, mostly concentrated south of the Yinshan and east of the Daxingan Mountains. Concomitantly, the southern areas are mainly agricultural, and the population is mainly of Han ethnicity, following the traditional farming civilization. The northern region contains mostly grassland pastoral areas, and the nomadic culture of the Mongolian “living by water and grass” has affected these areas [31]. Although grasslands were contracted to households in Inner Mongolia after the founding of New China, the lifeways of herdsmen gradually changed from nomadic to semi-settled, and then to settled, but a certain land area is required for their lifestyle [51]. Driven by the increase in population and economic interests, the production enthusiasm of farmers and herdsmen has been mobilized, and the number of sheep has increased significantly [52]. Transitional grazing and arbitrary reclamation have aggravated grassland degradation, and herdsmen have begun to relocate, thus affecting the spatial pattern of settlements. The arid climate and severe water shortages in the north also restrict the pattern of rural settlements, and this phenomenon is most prominent in the Xilingol grassland [31, 52].

This study confirms the findings of earlier research [2], that rural settlements in the cold alpine region have a clustering pattern at low density. More specifically, this study found that rural settlements in the eastern part of Inner Mongolia have the lowest kernel density but the highest degree of aggregation. The winter in the northeast is long and cold [53]. The average temperature of the coldest month (January) is below −24°C and the extreme minimum temperature is about −50°C, in line with the cold continental monsoon climate. The temperature is also the most critical factor affecting rural settlements in eastern Inner Mongolia. This phenomenon has also been confirmed in the early studies [18], which pointed out that in the cold region of northeast China, temperature determines the growth of crops, and rural settlements are greatly affected by the distribution of cultivated land. The degree of agglomeration of rural settlements in the west is low, because this region is located inland, which is arid and has little rain, and the ecological environment is fragile [41, 42, 51]. Under the coercion of human disturbance and climate change, the ecological environment has deteriorated rapidly, drought has intensified, vegetation has degraded, and desertification has expanded, resulting in low land production capacity. Consequently, the impact of vegetation types and coverage has increased on the spatial distribution of residential areas in the region, but the explanatory power of the population density factor has significantly declined. Although the effects of different factors on rural settlements are different for different regions and their interactions are not consistent. It is undeniable, however, that cultivated land, towns, and population density factors are the most important in Inner Mongolia. Numerous studies have shown that, in traditional farming societies, cultivated land is the basic guarantee for rural development, and villages are distributed close to cultivated land [16, 17]. This is the case in Inner Mongolia, which is a large agricultural province, and the importance of cultivated land is irreplaceable. With the development of the economy and the acceleration of urbanization, the influence of central towns has become more prominent [7, 9, 28, 32]. Population density has an impact not only on the distribution of rural settlements, but also on local economic development, resource allocation, and other important effects [34, 36]. The data from the seventh census show that the population of Inner Mongolia has further concentrated in economically developed regions, urban agglomerations, and capital cities; the size of rural households continues to shrink, with some villages disappearing as a result; and the aging of the population is further intensifying, which is a worrying phenomenon. A study found that senior peoples have played a leading role in driving up GHG emissions over the past decade and are becoming the largest contributor [54].

From 1990 to 2020, the rural areas of Inner Mongolia, like other regions in China, experienced a population decrease and land use increase. In recent years, Inner Mongolia has urbanized rapidly, and the high-quality educational resources, medical conditions, sound infrastructure, and more employment opportunities in the city have attracted rural young people [7, 55]. However, due to the influence of the household registration system, social relations, and hometown nostalgia, the roots of these people remain in rural areas [24, 38]. With the increase in economic income, they tend to return to their hometown to build houses [4, 22, 23]. As the people grow older, their labor ability is weakened, and their willingness to return to their hometown increases [40, 43, 56]. This phenomenon needs further scientific response.

Upon the New Urbanization Plan of the Inner Mongolia Autonomous Region issued by the Inner Mongolia government in 2021, the urbanization rate for the permanent population in Inner Mongolia will reach about 69% by 2025, and 72% by 2035 (https://www.nmg.gov.cn/). It indicates that an increasing proportion of the rural population will enter urban areas, and the problems of urban expansion and rural recession will become more serious. Urban expansion primarily encroaches on cultivated land [25–27], which seriously threatens national food security [49]. As the basic resource of agricultural production, cultivated land is indispensable in both traditional agricultural society and modern economic society. However, excessive curbing of the process of urbanization will also have a negative impact on the Great Rejuvenation of the Chinese Dream, which requires comprehensive coordination. It is worth affirming, however, that the optimization and regulation of rural areas must be carried out according to local conditions, especially in the minority border areas of Inner Mongolia. The willingness of local residents should be assured, and the regional reality, national culture, customs, and religious beliefs should be respected because the previous government-led a top-down remediation plan or relied solely on the short-term design of planners—without the participation of local residents in the optimization of control—and has not been able to achieve better results [5, 9, 43].

Importantly, the expansion of urban land in Inner Mongolia is also encroaching on grassland and forest resources. Overgrazing, reckless reclamation, climate change, and human disturbance have led to serious degradation of grasslands and forests [44], which not only affects the regional ecological environment but also threatens biodiversity [41, 51]. In particular, the degradation of grassland and forest, as the main terrestrial carbon sinks, will have a negative impact on China’s double carbon strategy [49]. The small-scale and highly dispersed rural areas in Inner Mongolia do not have centralized heating. Each area mainly uses small coal-fired boilers for heating alone, and the quality of coal is not good. The exhaust generated by coal combustion is directly discharged at low altitudes [53], which further aggravates environmental pollution and has a negative impact on the double carbon target. Centralized clean heating is an important part of optimizing the rural living environment and realizing rural revitalization in the context of implementing the double carbon goal.

Inner Mongolia is in a new era of rapid economic development, and the urban and rural gap not only exists but also will continue to expand. A scientific response is therefore necessary to accelerate the process of urban and rural integration in Inner Mongolia. Drought is the main climatic characteristic of Inner Mongolia [41, 42, 44]; hence, it is necessary to acknowledge water resources as the greatest rigid constraint, insist on fixing the city, land, people, and production with water accurately and reasonably plan the development of the population and urban and rural industries; and take a sustainable and high-quality development path. In the context of the double carbon strategy, it is important to consider the carbon-carrying capacity as the basis to focus on regional territorial spatial planning and optimize the improvement of the rural habitat.

## 5. Conclusions

Inner Mongolia, China is a vast territory with a complex and diverse topography and a fragile ecological environment. It is also the frontier of ethnic minorities and a key area for rural modernization in the new era. Using the spatial distribution data of multi-decadal settlements, this study revealed the spatial distribution characteristics of rural settlements and influencing mechanisms in different periods, scales and regions.

The distribution of rural settlements in Inner Mongolia had obvious spatial differences. The Hetao irrigation area had the largest kernel densities, which increased first and then decreased. The central Hohhot and eastern Chifeng and Tongliao areas had higher kernel densities and showed an increasing trend throughout the study period. The northeastern Hulun Buir, the central Xilingol League, and the westernmost Alxa League had the lowest kernel densities. The spatial distribution pattern of rural settlements in Inner Mongolia was characterized by clustering, with the highest degree of agglomeration in the cold eastern region, followed by the central region, and the lowest level of agglomeration in the western region. Cultivated land, towns, and population density were important factors influencing the spatial distribution of rural settlements. The eastern region was mainly shaped by the temperature, and vegetation type and cover played a greater role in influencing the western region. The interaction between two influencing factors on the spatial distribution of rural settlements was enhanced. The rural areas of Inner Mongolia also experienced the phenomenon of population decrease and land use increase, which became more prominent in the last decade. The expansion of rural areas in Inner Mongolia encroached on cultivated land, grassland, and unused land; the settlement conversion into cultivated land and grassland also occurred, but in low proportion. There is much room for improvement in the optimization and control of rural habitats in Inner Mongolia. We used the spatial distribution data of rural settlements with a resolution of 1 km to study the spatial distribution pattern and influencing mechanism of rural settlements in the eastern, central and western regions of Inner Mongolia at the regional scale. Although the time span is about 30 years, it is relatively macroscopic. The selection of influencing factors does not take into account factors such as policy system and national culture, and there are certain deficiencies. Applications of medium and high-resolution remote sensing images, such as sentinels, have facilitated their research at microscales. The follow-up study should be based on high-resolution images, integrate policy systems and other factors, explore the optimization layout of rural settlements in Inner Mongolia under the background of dual carbon, and promote the high-quality development of rural areas in Inner Mongolia.

## Supporting information

S1 Data (ZIP)
